# Cytomegalovirus colitis in immunocompetent hosts: A case report and literature review

**DOI:** 10.1002/ccr3.8435

**Published:** 2024-01-08

**Authors:** Mohammadreza Salehi, Nahid Shafiee, Maryam Moradi

**Affiliations:** ^1^ Department of Infectious Diseases Imam Khomeini Hospital, Tehran University of Medical Sciences Tehran Iran; ^2^ Eye Research Center, The Five Senses Health Institute Rassoul Akram Hospital, Iran University of Medical Sciences Tehran Iran

**Keywords:** CMV, CMV colitis, cytomegalovirus, immunocompetent, rectal bleeding

## Abstract

**Key Clinical Message:**

Rectal bleeding can manifest cytomegalovirus (CMV) colitis even in immunocompetent patients, which can be cured with ganciclovir treatment.

**Abstract:**

Cytomegalovirus (CMV) is an opportunistic virus widely affecting immunocompromised patients. Different manifestations varied from asymptomatic in immunocompetent individuals to end organ involvement, such as colitis in those with immunodeficiency. Despite the rarity of CMV colitis in immunocompetent hosts, we should consider it when the other conditions have been excluded. In this article, we have described a case of CMV colitis in an immunocompetent host and have performed a literature review on this entity. An immunocompetent 70‐year‐old female was admitted to the hospital with recurrent rectal bleeding. After various evaluations including laboratory analysis, stool examination, and colonoscopy, we have detected superficial lesions. Pathology and polymerase chain reaction reports favored CMV involvement. Her condition continues to improve after intravenous ganciclovir infusion. Rectal bleeding can manifest CMV colitis even in immunocompetent patients, which can be cured with ganciclovir treatment.

## Graphical abstract

Cytomegalovirus colitis in an immunocompetent host.

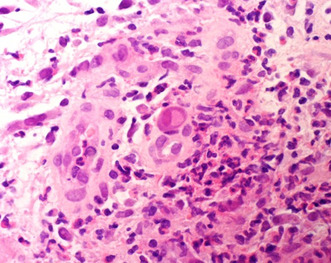

## 1 BACKGROUND

Cytomegalovirus (CMV), a double‐stranded DNA virus, is a member of the Herpesviridae family. It has the largest genome among all viruses, which can affect humans. In people with a healthy immune system, CMV infection causes a self‐limiting, asymptomatic infection; in immunocompromised patients, it can be complicated.^1^ CMV can infect almost all adults and is one of the main causes of infections in people who have had transplants. It affects about 5%–15% of patients, even with various efforts to prevent it.^2^ In Iran, the number of babies born with congenital CMV ranges from 0.2% to 2%, with an average of 0. 65%.^3^

Depending on the host condition, CMV infection can have a wide range of symptoms and complications, from an asymptomatic infection in healthy people to end organ damage in those with immunodeficiency.^4^ Typical clinical manifestations of primary CMV infection include fever, malaise, myalgia, arthralgia, and anorexia which can be similar to other conditions such as mononucleosis and COVID‐19.^5^, ^6^ CMV reactivation in immunocompromised patients can lead to gastrointestinal tract problems such as inflammation or hemorrhage and hematologic abnormalities such as leucopenia, thrombocytopenia, and lymphocytes.^7^

Known immune deficiencies associated with serious CMV diseases include disorders in all or parts of the immune system after transplantation, immunosuppressive therapy, and immunosuppressive diseases such as AIDS. Common end organ disorders caused by CMV in immunocompromised patients include hepatitis which can lead to fulminant liver failure, retinitis especially in HIV‐positive patients, colitis and esophagitis as the first and second most common gastrointestinal tract manifestation of CMV, pneumonitis, especially in hematopoietic stem cell transplant recipients, rarely transverse myelitis, and even encephalitis with poor prognosis.^8^, ^9^, ^10^, ^11^, ^12^, ^13^, ^14^

CMV colitis usually occurs in immunocompromised patients and is prevalent from 21% to 34% worldwide. The symptoms of CMV colitis are very similar to inflammatory bowel disease (IBD) colitis exacerbation.^15^, ^16^

Several methods such as serological testing, endoscopic evaluation, tissue biopsy, real‐time PCR CMV DNA quantification, and viral culture, exist to identify CMV infection.^9^ Endoscopic specimens during colonoscopy examination from the intestinal ulcers are needed to identify the histological diagnosis of CMV colitis. Real‐time PCR and viral culture have high sensitivity and specificity for CMV colitis diagnosis while the long duration delayed the diagnosis and treatment.^16^

It is uncommon for people with a healthy immune system to get CMV colitis. Some variables are consistent with immunocompetent patients with CMV colitis including male gender, advanced age, and other comorbidities such as diabetes. In those without the mentioned variables, a good prognosis and full recovery are expected.^8^ Due to the severe side effects of antivirals, treating CMV colitis is limited, even in people with immunodeficiency. Therefore, ganciclovir is given to prevent future severe complications.^9^, ^17^

According to the literature review, we expect to see CMV colitis in immunocompromised patients, but there were several immunocompetent hosts with the same condition. In this article, we have described a case of CMV colitis in an immunocompetent host and have described the diagnostic approach, management, and clinical course in such cases.

## 2 CASE PRESENTATION

A 70‐year‐old woman with a history of severe anemia and well‐controlled diabetes was admitted with recurrent rectal bleeding, low‐grade fever, diarrhea, and abdominal pain. The patient primary laboratory data include white blood cell count of 8.1 × 109/L (normal range of 4.5–11.0 × 109/L), absolute neutrophil count of 6200 (normal range of between 2500 and 6000), hemoglobin of 10.2 g/dL (normal level for females is 12–16 g/dL), platelet count of 210 (normal range of 150,000–450,000 platelets per microliter of blood), creatinine level of 1.08 (normal range of 0.6–1.1 mg/dL for women), alanine aminotransferase (ALT) level of 21 (normal range of 19–25 IU/L for females), aspartate aminotransferase (AST) level of 18 (normal range of 10–36 units/L for females), and positive stool culture tests for Clostridium difficile enteritis.

**FIGURE 1:**
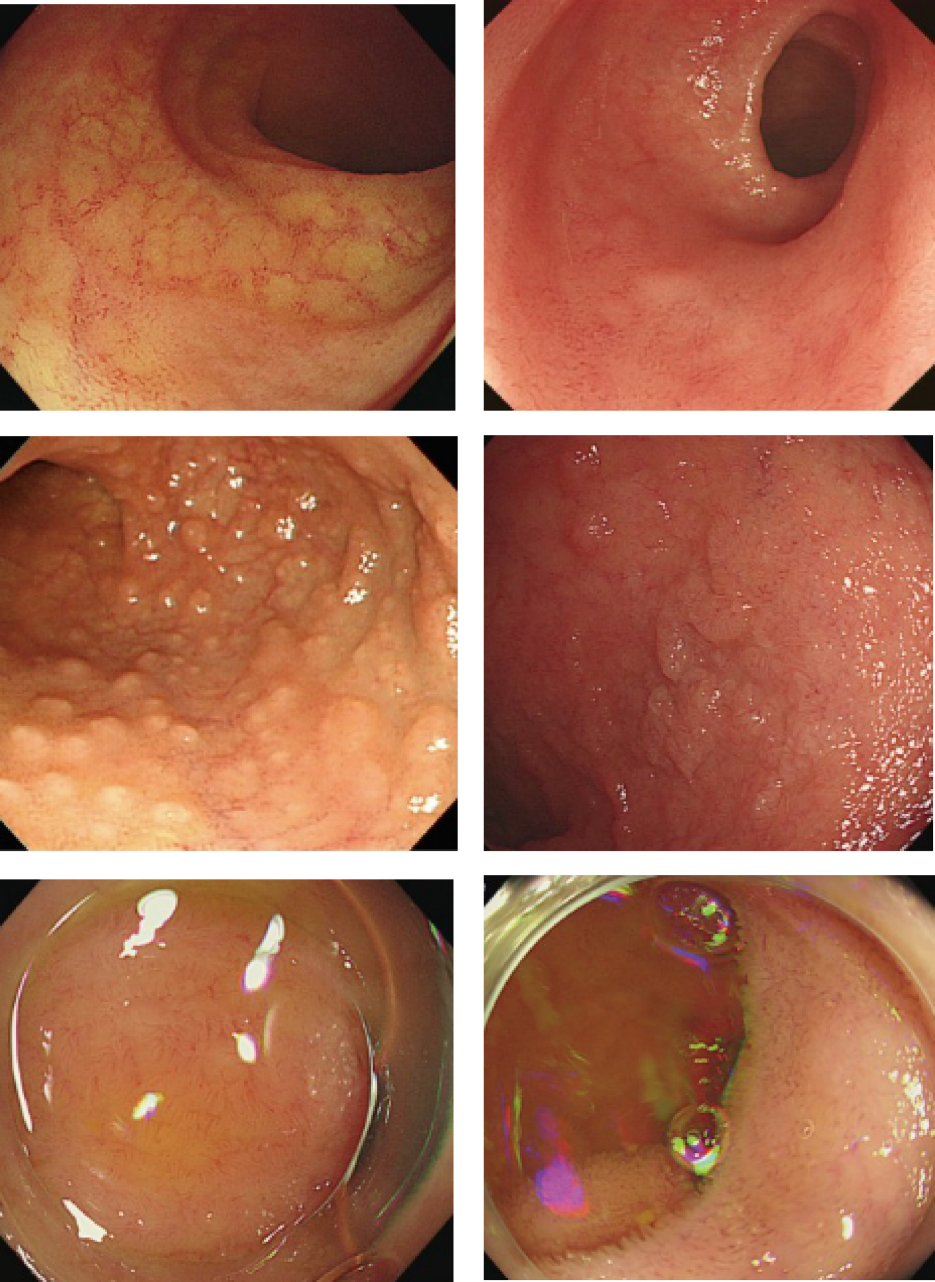
Patient's colonoscopy images consist of mucosal inflammation due to cytomegalovirus colitis.

**FIGURE 2:**
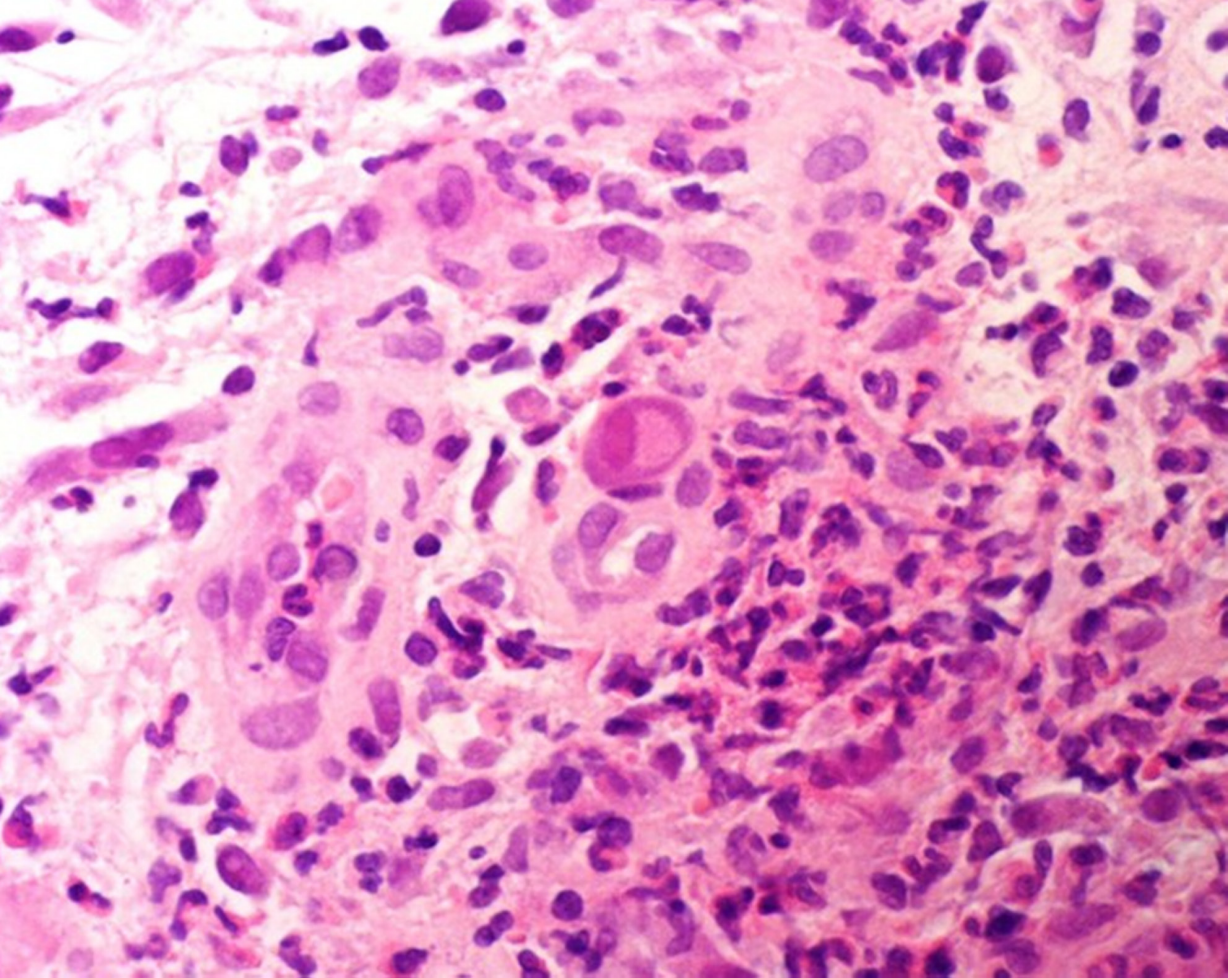
Patient's mucosal tissue sample indicates cytomegalovirus infection.

Her bleeding continued during hospitalization despite extensive antibiotic treatment against Clostridium difficile. We have done laboratory evaluations and colonoscopies (Figure 1). We detected superficial clone lesions during a colonoscopy, confirmed as CMV colitis, after polymerase chain reaction (PCR) and pathological tests (Figure 2).

We have initiated a 5 mg/kg intravenous (IV) ganciclovir infusion every 12 h for a week. The patient's symptoms have started to improve after the IV ganciclovir infusion. The patient was discharged with valganciclovir 900 mg PO q12 h for 21–42 days after 1 week of hospitalization. The treatment was changed from ganciclovir to valganciclovir based on our patient past medical history of severe anemia and the potential side effect of ganciclovir in decreasing bone marrow function.

## 3 LITERATURE REVIEW

A literature search was performed from 1998 to 2021 using the computerized PubMed database. English‐available articles considering CMV colitis in immunocompetent hosts were reviewed. The terms “CMV colitis” AND “immunocompetent” were explicitly sought. All types of studies dealing primarily with CMV colitis were reviewed, and those with any immunodeficiency were excluded. The exclusion criteria were any congenital immune deficiency, transplantation, HIV infection, chemotherapy, steroid therapy, any malignancy, and other comorbidities such as diabetes or renal failure.

Despite the high prevalence of CMV colitis in immunocompromised individuals, this involvement is rare in those with adequate immunity. According to studies in this field, immunodeficiency can increase the risk of developing CMV colitis even to a minimal extent; pregnancy, kidney failure, malignancy, and diabetes are comorbidities that can lead to developing this complication. Aging (above 55) is another condition that can increase the rate of CMV colitis. Therefore, CMV colitis in people with appropriate immunity is rare.^8^, ^18^

Diarrhea following CMV colitis is rare in the elderly and healthy children as opposed to younger people. However, we should consider CMV colitis in those who present to the hospital with prolonged diarrhea; other tests do not reveal a diagnosis.^19^, ^20^, ^21^

CMV colitis often involves the colon and the rectum in non‐immunocompromised hosts. Its manifestation can be rectal bleeding, which is similar to the immunocompromised patients. For proper diagnosis, a complete colonoscopy with several biopsies is needed.^22^ Based on other studies, CMV colitis can present with pneumonia‐like symptoms such as productive cough, fever, chills, and anorexia, along with other abdominal symptoms. Therefore, we should consider CMV colitis in unexplained abdominal symptoms.^23^

Intestinal manipulation and disruption for various reasons, such as bariatric surgery or conditions like amebiasis or shigellosis, with the mechanism of damage to the intestinal mucosa and malnutrition, can predispose healthy people to CMV colitis.^24^, ^25^, ^26^

According to a retrospective study on clinically ill patients with several comorbidities in the intensive care unit (ICU), despite the very low prevalence of CMV colitis in immunocompetent individuals, we should consider this uncommon complication in case of encountering lower abdominal bleeding in the ICU setting.^27^

CMV colitis was reported in a healthy older adult (82 years) presenting with bloody diarrhea. According to the abdominal pain and the patient's age, ischemic colitis was first diagnosed. Due to the continuation of the patient's symptoms, a diagnostic sigmoidoscopy was performed with a biopsy. According to the pathology report, based on the observation of CMV inclusion bodies, the patient was diagnosed with CMV colitis, and recovery was achieved after starting treatment with ganciclovir.^28^

CMV colitis atypical presentation was reported in a healthy individual in 2020. He was admitted several times with vigorous symptoms such as weight loss, poor appetite, abdominal discomfort, and other constitutional symptoms. He then progressed to sepsis and lactic acidosis in later admission. With the diagnosis of IBD exacerbation, the patient was treated with steroids and mesalamine. Due to the lack of recovery and worsening of clinical condition, the patient underwent a diagnostic laparotomy. Based on the pathology response, the patient was diagnosed with ischemic ileocolitis with superimposed CMV colitis. According to the diagnosis, treatment with ganciclovir was started. Finally, the patient was discharged with a favorable general condition and oral ganciclovir.^29^ Another atypical presentation was a CMV‐associated tumor at the ileocecal valve of the cecum. After less than 2 months, the patient's clinical symptoms, such as abdominal pain and laboratory tests, were typical. We have not observed any other lesions in later evaluations. Therefore, CMV‐associated pseudotumor should always be considered a differential diagnosis of colon lesions.^30^ We have summarized the list of immunocompetent hosts with CMV colitis in Table 1.

**TABLE 1:** Immunocompetent patients with cytomegalovirus colitis (based on a literature review).

Age	Sex	Symptoms at admission	Site of involvement	Year
68	Male	Chronic diarrhea	Ileocolitis	2020^29^
8‐week‐old	Male	Watery diarrhea	Diffuse in all colon	2016^19^
10‐month‐old	Male	Bloody diarrhea and fever	Diffuse in all colon	2016^19^
29	Male	Acute confusional state	Diffuse in all colon	2011^23^
61	Female	Productive cough	Rectum and sigmoid	2011^23^
76	Male	Abdominal pain	Ascending colon and cecum	2010^30^
64	Female	Diarrhea and nausea	Rectum	2006^20^
82	Male	Diarrhea and abdominal pain	Sigmoid and rectosigmoid	2005^28^
2‐Month‐old	Male infant	Diarrhea and severe dehydration	Sigmoid	2004^25^

## 4 DISCUSSION

CMV, as a member of the Herpesviridae family, can cause various diseases in humans depending on their underlying immunity. Despite the high mortality in patients with underlying immune deficiency, the disease manifests itself with symptoms similar to mononucleosis in immunocompetent patients. Several case reports show CMV can cause single‐organ damage even in patients with intact immunity.^31^ Based on previous studies, CMV colitis can occur in immunocompetent and immunocompromised and IBD patients.^32^

Although we discovered that CMV colitis and intestine inflammation can happen in patients with impacted immune systems, it is still uncertain if these patients are immunocompetent. Many patients had advanced age and long‐lasting health problems such as diabetes and chronic kidney disease. It has been suggested that CMV colitis is rare in those with actual immunocompetency who are young and without any comorbidities.^33^ In immunocompetent patients, CMV colitis confirmation is based on histological and pathological examinations. A colonoscopy is required to have a complete vision of existing lesions and to prepare biopsies for further evaluations. There is no characteristic lesion for CMV colitis.^19^ In our patient, we detected superficial lesions, and the histological examination revealed CMV colitis.

After the diagnosis, ganciclovir treatment is required to eradicate the virus and improve the patient's complications.^31^ Our patient responded slowly to the ganciclovir treatment, and her symptoms improved during 1 week of hospitalization.

Based on our study, CMV colitis should be considered in patients with rectal bleeding when other explanations were ruled out, even those with intact immunity. Due to the full recovery after ganciclovir treatment, diagnosing the case before exacerbation is essential. In conclusion, because CMV colitis is less prevalent in immunocompetent people, it would seem that immunocompetent people are less susceptible than immunocompromised people.

## 5 CONCLUSION

Immunocompetent patients can be vulnerable to CMV colitis, similar to those with immune deficiencies; therefore, in patients with rectal bleeding and non‐responsible antibiotic therapy, CMV colitis should be considered. Future studies on more CMV colitis patients in multiple medical centers can help distinguish the characteristics of CMV colitis in immunocompetent patients clearly.

## AUTHOR CONTRIBUTIONS

**Mohammadreza Salehi:** Conceptualization; supervision. **Nahid Shafiee:** Resources. **Maryam Moradi:** Data curation; methodology; writing – original draft.

## FUNDING INFORMATION

None.

## CONFLICT OF INTEREST STATEMENT

The authors declare that they have no competing interests.

## CONSENT

The author confirms that written consent has been obtained from the patient for submission and publication.

## Data Availability

The authors confirm that the data supporting the findings of this study are available within the article.
